# Christian Science

**Published:** 1908-07-25

**Authors:** Frederick Dixon

**Affiliations:** 23 and 24 Clun House, Surrey Street, Strand, W.C.


### EDITOR'S LETTER-BOX.

CHRISTIAN SCIENCE.

Dear Sir,—Will you permit me to refer in a few words to the, I venture to think, quite unnecessary remarks on Christian Science, which the Bishop of London introduced into his address to the Medical School of St. Mary's Hospital, mentioned in your issue of July 11?

Christian Science would appear to be fast assuming, in his Lordship's public utterances the place which King Charles's head occupied in Mr. Dick's memorial to the Lord Chancellor; but that is scarcely a reason why he should emphasise an antagonism between Christian Scientists and medical men, which has absolutely no existence within the Christian Science movement.

First of all, there is no need whatever for the Bishop to come to the assistance of the medical profession, which already represents the big battalions; and, secondly, every Christian Scientist, so far from feeling any antagonism towards doctors, has for them the same admiration as for all men who, whatever their differences, are earnestly striving to overcome the sorrow and misery of the world in the way that seems truest to them. For themselves they have found, they believe, a more excellent way, a way which brings healing not only to those suffering from disease and pain, but to those in sorrow, in want, and in despair. This to their understanding, is the healing so beautifully described by Whittier in the words:

"The healing of the seamless dress
Is by our beds of pain;
We touch Him in life's throng and press,
And we are whole again."

Yours truly,

Frederick Dixon.

23 and 24 Clun House,
Surrey Street, Strand, W.C.,
July 16, 1908.

[We give Mr. Dixon's letter the same publicity that we afforded to the Bishop's remarks: though we must say we find ourselves in considerable sympathy with Dr. Ingram. Probably both the latter and the medical profession would be less opposed to what is known as Christian Science if all its advocates were as fair-minded and moderate as Mr. Dixon. Ed.]

